# Commentary: Case report: Primary intraosseous poorly differentiated synovial sarcoma of the femur

**DOI:** 10.3389/fonc.2022.1095399

**Published:** 2023-01-04

**Authors:** Jiro Ichikawa, Hiroki Imada, Satoshi Kanno, Tomonori Kawasaki

**Affiliations:** ^1^ Department of Orthopaedic Surgery, Interdisciplinary Graduate School of Medicine, University of Yamanashi, Yamanashi, Japan; ^2^ Department of Pathology, Saitama Medical Center, Saitama Medical University, Saitama, Japan; ^3^ Department of Pathology, Saitama Medical University International Medical Center, Saitama, Japan

**Keywords:** synovial sarcoma, differential diagnosis, primary site, bone metastasis, rare case

## 1 Introduction

We read with great interest the article by Dr. Pang and colleagues entitled “Case Report: Primary Intraosseous Poorly Differentiated Synovial Sarcoma of the Femur,” published in *Frontiers in Oncology* (1). The authors concluded that the primary site of poorly differentiated synovial sarcoma (SS) in this patient was not soft tissue, but bone, and that misdiagnosis between SS and Ewing sarcoma (ES) had occurred. However, we had some concerns regarding the interpretation of this unusual case, which we would like to discuss with the authors.

## 2 Primary site diagnosis

Our first concern regards whether the primary site of this tumor was the bone or soft tissue.

Case summary: Magnetic resonance imaging (MRI) showed two tumors; one was located in the intraosseous lesion and the other in the deep soft tissue between the adductor muscles of the thigh. The intensity of T1- and T2-weighted images of the intraosseous tumor was almost the same as in the soft tissue tumor. These tumors seemed not to be connected but adjacent because the tumor in the soft tissue was surrounded by muscle and fat. The size of both tumors was >5 cm. Positron emission tomography-computed tomography (CT) revealed slight uptake in the two tumors and a pathological fracture of the thigh. CT also revealed multiple nodules in the lung. The candidates for the primary site at diagnosis were the bone, soft tissue, and lung. Approximately 70% of SS occur in the deep soft tissue of the lower and upper extremities (2); in pediatric cases, 82% are located under the fascia and 70% in the lower extremities (3). The bone, lung, peripheral nerves, etc., have been reported as unusual primary sites (2). There are 13 cases reported as intraosseous SS confirmed by fluorescence *in situ* hybridization (FISH) or polymerase chain reaction (4–14). The ages of the patients were >20 years and the most frequently affected bone was the tibia (5 cases). Among the 10 cases in which metastasis at diagnosis was discussed, there were 8 with no metastasis and 2 with lung metastasis. Although the lung is well known as a metastasis site (2, 15, 16), there was a case with primary lung SS metastasized to bone (17); therefore, special caution is required when two separate tumors exist at diagnosis. In cases in which two tumors coexist, it is difficult to completely distinguish between the primary and metastatic sites using histopathological and imaging findings. The primary site of this case was determined with no metastasis at diagnosis. Although the authors stated that osseous metastases in soft tissue malignancy tend to be multiple, in the SS cases with bone metastasis, 33% are reported as solitary and 67% as multiple (16), suggesting that solitary bone metastasis of SS is not uncommon. The most common metastatic site of SS is the lung at 70–80%, followed by the bone and lymph nodes (2, 15, 16). Considering these findings, we strongly question whether the SS in the soft tissue had perhaps metastasized to the bone and lung.

## 3 Time for metastasis

Our second concern is that the authors state that the time for metastasis was short; in this case, the pain was derived from the bone lesion with the pathological fracture, suggesting that the duration of the skeletal-related event (SRE) was 7 months. There is a possibility that the soft tissue lesion was in existence prior to this because in pediatric cases, painlessness and lack of palpability are distinctive clinical features (3); in this case, both symptoms were present and there was no complaint of the soft tissue lesion. The median time to SRE in bone metastasis patients affected by soft tissue sarcoma is 1–9 months (average 4 months) and the time course is similar to this case (18). SS generally grows slowly and the duration of clinical symptoms is as long as 2–4 years (3, 15). Of course we will never know when these two tumors first occurred, but considering these findings, there is a chance that the soft tissue lesion might have occurred prior to the 7-month period.

## 4 Misdiagnosis as ES

Finally, one thing that occurred which might not be directly related to the primary site was the misdiagnosis as ES. Recently, in addition to Ewing family tumors, round cell sarcomas with EWSR1 gene fusion, BCOR-rearrangement, and CIC-rearrangement have been recognized as new entities; furthermore FISH and PCR are essential for the differential diagnosis between ES and Ewing family tumors (19). Even if misdiagnosis had not occurred, we imagine that there would have been little difference in prognosis because the case involved a tumor that was >5 cm in size, metastasis at diagnosis, the poorly differentiated variant were an unfavorable factor (15). 

## 5 Conclusion 

In conclusion, we believe that this typical SS originated from the deep soft tissue and metastasized to the bone and lung. In the case of tumors within multiple organs, it is often difficult to establish which is the primary site and careful analysis is required, especially when concluding rare events.

## Author contributions

Conception/design: JI, HI, and TK. Provision of study material or patients: JI, HI, and TK. Data collection and analysis: all authors. Manuscript writing: JI, HI, and TK. All the authors read and approved the manuscript.
